# A new treatment of concealed penis: symmetrical pterygoid flap surgery

**DOI:** 10.1590/S1677-5538.IBJU.2023.0629

**Published:** 2024-02-07

**Authors:** Peng Jing, Dan Zhao, Qiao Wu, Xiaohou Wu

**Affiliations:** 1 Urology surgery of the First Affiliated Hospital of Chongqing Medical University Chongqing China Urology surgery of the First Affiliated Hospital of Chongqing Medical University, Chongqing, China;; 2 Pediatric Surgery of the Affiliated Hospital of North Sichuan Medical College Sichuan Nanchong China Pediatric Surgery of the Affiliated Hospital of North Sichuan Medical College, Sichuan Nanchong, China

**Keywords:** Penis, Surgical Flaps, Therapeutics

## Abstract

**Purpose:**

Considerable controversy exists regarding the surgery for concealed penis. We describe a new technique for repairing concealed penis by symmetrical pterygoid flap surgery.

**Methods:**

From January 2016 to July 2022, we evaluated 181 cases of concealed penis that were surgically treated using the symmetrical pterygoid flap surgery. We measured the penile size preoperative and 2, 4, 12 weeks, and 1 year postoperative to confirm the improvement. A questionnaire was administered to the patients and parents to assess satisfaction regarding penile size, morphology, and hygiene.

**Result:**

The perpendicular penile length was1.59±0.32cm preoperative and 3.82±1.02 cm after the procedure (p < 0.05), and 4.21±1.91cm after one year of postoperative (p < 0.05). The overall satisfaction of patients was 97.89%, while the overall satisfaction of older children patients (age>7) was 75.24%. Parents focus more on the penile exposure size, while patients focus more on the penile morphology. Almost every patient had postoperative penile foreskin edema. However, this symptom had spontaneously resolved by 4-6 weeks. The complications such as skin necrosis, tissue contracture, or wound infection were 4.42%.

**Conclusion:**

The symmetrical pterygoid flap surgery is an effective surgical technique for the management of concealed penis in children producing predictable results and excellent satisfaction of the parents and patients.

## INTRODUCTION

**Figure 1:**
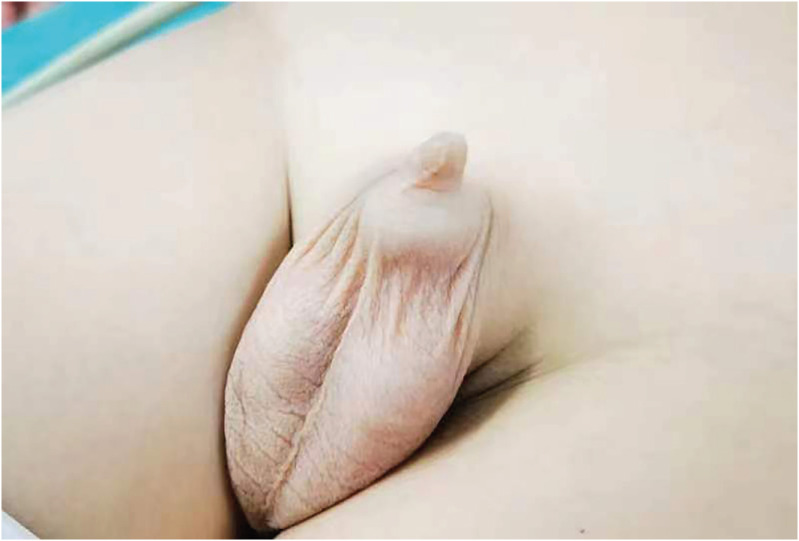
Picture of Concealed Penis before surgery.

Concealed penis refers to a poorly developed penis which is hidden in the subcutaneous tissue (1). The anomaly is specifically associated with a lack of adequate outer penile skin and inadequate subcutaneous attachment to Buck's fascia, due to which the penis seems to be fused with the scrotum (2). When the penis is not erected, it is often shaped like a beak, and its normal shape disappears (Figure-1). Concealed penis can cause phimosis, balanitis, and insevere cases may cause difficulty in urinating (3). If the penis is concealed until adolescence or adulthood, it is a significant cause of psychological trauma and sexual dysfunction in adolescents and adults (4, 5). Therefore, surgical treatment is necessary for patients with concealed penis, and it has been extensively promoted by urologists. It was reported that there are numerous surgical techniques for surgical treatment; however, the postoperative results obtained by different surgical methods are significantly different, which leads to no single surgical method being widely adopted (2, 5, 6). One of the numerous surgical techniques for concealed penis, named symmetrical pterygoid flap surgery, is rarely reported. In our centre, some children with concealed penis were treated in this way and have achieved stunning postoperative results. Therefore, this study aims to share our experience and hope to provide some reference to the treatment of children with concealed penis.

## METHODS

### Patients and study design

From January 2016 to July 2022, we evaluated 181 cases of concealed penis that were surgically treated using the symmetrical pterygoid flap surgery. The clinical manifestation of all cases was as follows: the appearance of the penis looks like a "bird's beak", the penis appears to fuse with the scrotum and the penile shaft is entrapped within the subcutaneous tissue, and the normal penile shaft could be palpated while applying pressure on the opposite side of the shaft base (5-7). All patients were scheduled for regular follow-up visits at 2, 4, 12 weeks, and 1 years postoperative. Data were collected and analyzed on the patient's preoperative penile length, postoperative penile length, penile circumference, and postoperative complications. At last, patients' and their parents' expectations and satisfaction about the surgery were also collected and analyzed.

### Surgical Technique

**Figure 2:**
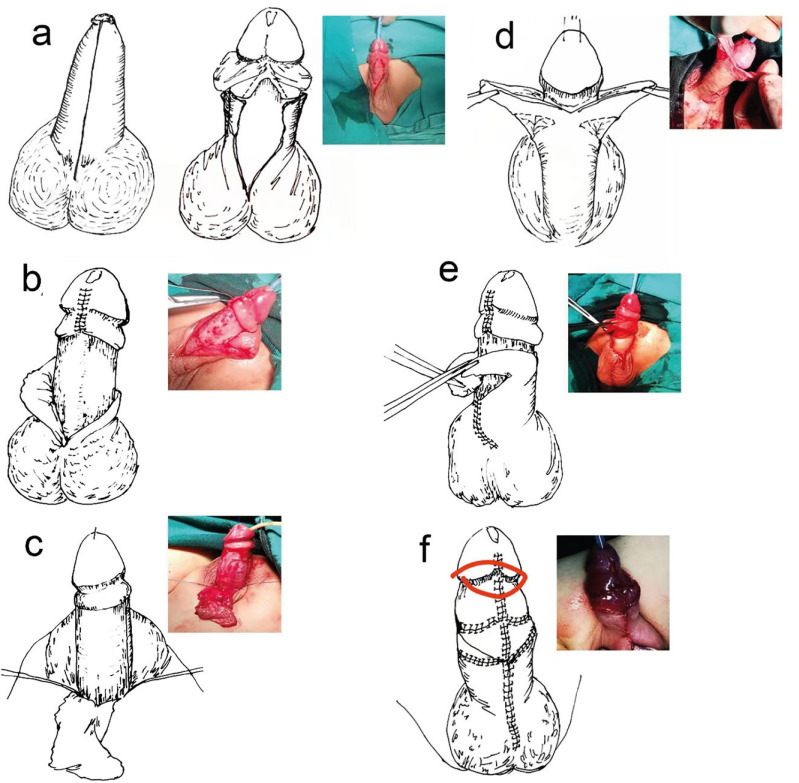
Diagram of surgical steps and Intraoperative picture.

**Figure 3:**
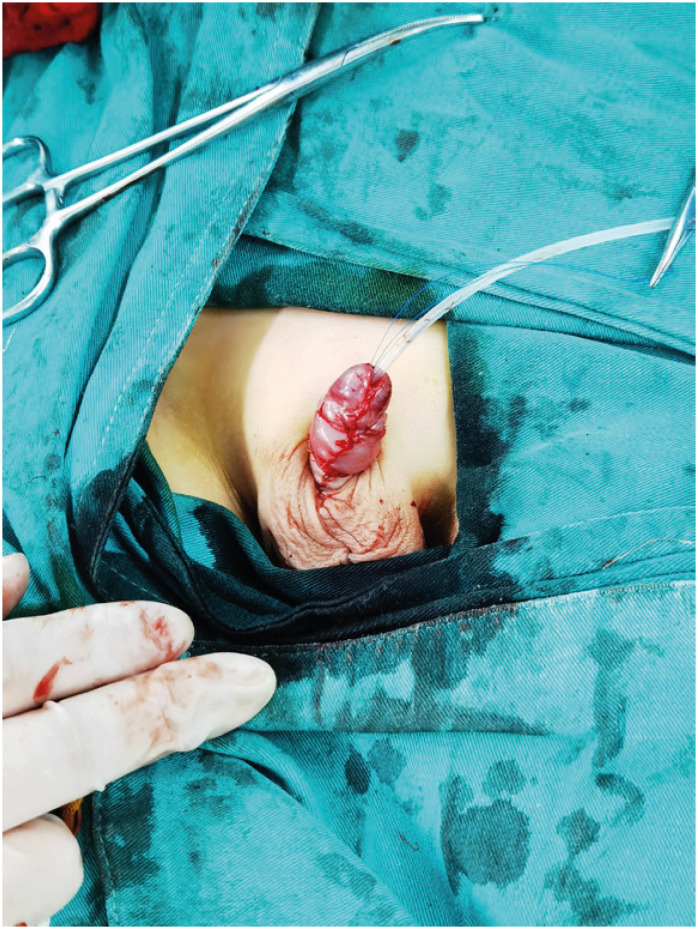
Postoperative picture of Concealed Penis.

All surgical procedures were carried out by a single urologist. The procedures were conducted under general anesthesia with patients in the supine position. The critical surgical steps were as follows. First, a longitudinal skin incision was performed at the mid-ventral aspect of the prepuce, from the penile scrotal junction section to the proximal to the coronary sulcus, releasing of the stenotic ring and exposure of the glans (Figure-2a). Then, the penis is given control and pulled. At a distance of about 0.5 cm from the coronary sulcus, a circumcision at the inner prepuce was performed, and circumferential dissection along Buck's fascia freed the penis from its deep tethering to the penile base. Removal of the dysplastic and shrunk frenulum through an inverted V-shaped incision, and dissecting the inner prepuce around of foreskin frenum, lengthened the foreskin frenum by anastomosis (Figure-2b). Penoscrotal angles were reconstructed using 4-0 silk thread sewed between the tunica albuginea corporis sponge and dermis at the penile base, placed in the 10 and 2 o'clock positions (Figure-2c). The dorsal foreskin was longitudinally and symmetrically cut, forming two pedicled skin flaps. The subcutaneous fascia and vessels should be preserved, and the deep branches of the superficial vascular should be kept (Figure-2d). We then transferred the two pedicled skin flaps to the penile ventral area and inosculated the original foreskin. The two pedicled skin flaps were respectively connect with the ventral foreskin (Figure-2e). The superfluous inner plate of the penis was resected, and the foreskin was reconstructed with midline symmetric anastomosis on the ventral side of the penis (Figure-2f). The postoperative appearance of the penis was satisfactory (Figure-3).

### Statistical Analysis

All statistical analyses were performed using SPSS, version 20.0 for Windows (SPSS Inc., Chicago, IL, USA). All data were presented as mean ± standard deviation. Postoperative data were compared with preoperative values using the t-test. P-values lower than 0.05 were considered statistically significant.

### Ethical statement

Ethical approval was obtained from the Ethics Committee of Affiliated Hospital of North Sichuan Medical College (2022112412). The clinical operations involved in this article were evident to the children's parents. The children's parents signed the informed consent and agreed to disclose all the data.

## RESULTS

A total of 181 patients underwent the symmetrical pterygoid flap surgery for the concealed penis during the study period. The mean age of the patients in this study was 8.48±2.57 years, and the mean follow-up period was 35.67±17.73 months. The average operation time was 46.12±5.6 minutes. There was a statistically significant increase in penile length, from1.59±0.32 cm to 3.82±1.02 cm after the procedure (p <0.05), and to 4.21±1.91 cm after one year of postoperative (p < 0.05). But the circumference of the penis was increased from 4.78±0.65 cm to 5.88±1.11 cm (p>0.05), and the width of the glans was increased from1.27±1.12 cm to1.46±0.19 cm after one year of postoperative (p>0.05), and the results were not statistically significant (Table-1a).

**Table 1:** Penile size changes and satisfaction of postoperative.

Table-1a					
	Preoperative	Postoperative			
		2nd week	4 months	12 months	1 year
Penis length	1.59 ± 0.32	3.82 ± 1.02	3.80 ± 0.99	3.93 ± 0.99	4.21 ± 1.91
Glans width	1.27 ± 1.12	1.27 ± 1.12	1.31 ± 1.12	1.34 ± 0.14	1.46 ± 0.19
Penis circumference	4.78 ± 0.65	5.88 ± 1.11	5.60 ± 0.95	5.67 ± 0.95	5.91 ± 1.02

Table-1b			
		Parents (n=181)	Patients (n=164)
Focus point of postoperative	Size	43.75%	17.40%
	morphology	15.62%	52.17%
	Both	40.63%	30.43%
Recovery process		97.89%	75.24%
Overall satisfaction		97.89%	75.24%

All patients had postoperative penile foreskin edema, which resolved within four weeks. There were no complications, such as voiding difficulty or erectile dysfunction. Complications were noted in 8 patients and consisted of infection and dehiscence of skin on the ventral side of the penis (5 patients), penile skin flap necrosis (2 patients), scar hyperplasia of the penis (1 patient).

The postoperative satisfaction scale was used to investigate the satisfaction degree of the parents and the older children patients (Supplementary materials). The parents were more concerned about the exposed penile size (p < 0.05). Besides, they were also concerned about the penile morphology. When the size and the appearance couldn't be satisfied simultaneously, the parents chose the size rather than morphology(p < 0.05), but the older children patients chose the morphology rather than the size (p < 0.05) (Table-1b).

## DISCUSSION

Parents complain that their child's penis is small during the physical examination; however, a penis of normal size can be palpated in most of these boys [6].

The small size of the penis is only on appearance, but the penis is of normal size. The small appearance of the penis is named concealed penis. Concealed penis refers to a poorly developed penis, which is hidden in the subcutaneous tissue (1). According to the different causes, the concealed penis is divided into five subgroups: buried penis, trapped penis, hidden penis, inconspicuous penis and concealed penis (8, 9). Some concealed penis moderately retracted after release, and the phimosis and small penis should be excluded (10). The diverse causes of the concealed penis have been explored, including scarce outer penile skin, dysgenetic dartos fascia, poor skin fixation at the penile base, excessive suprapubic fat and inelastic fibrous bands (11-13). In addition to the poor display of the penis, we found the ventral foreskin was fused with the penile scrotum. All cases with severe concealed penis in this study presented with penile-scrotal fusion. In this study, we chose children with severely concealed penis. These children showed no significant pre-pubic fat accumulation, more than 1/2 of the penis body is hidden under the skin, the penis body is not exposed, and the appearance of the penis in an erect state is short conical, with penile-scrotum fusion.

Various surgical techniques have been described for the correction of the concealed penis. The key points of these procedures are removing excess pubic fat, releasing the dartos strip by peeling off the penis skin, anchoring the suprapubic skin to determine the penopubic angle, and repairing the shaft skin using various skin covering methods to correct the sparse skin shaft (1, 5, 6,13-16). Many surgical methods reported so far can effectively expose the penis. And the major difference in methods is which skin flap is used for the penile skin defect. This surgery has some limitations, like a poor release of the dartos fascia, lack of fixation at the penile base and poor esthetic results. The various surgical methods reported can increase the amount of penis exposure to a certain extent. However, for a severely concealed penis, the existing surgical methods can't fully satisfy the consideration of penis exposure and the aesthetic appearance of the penis.

We used a symmetrical pterygoid flap for covering the ventral skin defect after penile degloving during the surgical correction of the concealed penis. After observing a large number of normal children's penis, we have found a clear fusion line in the ventral centre of the normal foreskin, which is consistent with the left-right symmetry of the human body. If there is a deviation of the ventral midline of the foreskin, it is often combined with penile torsion in the clinic. The anatomical feature of the foreskin is that its superficial artery gives off a primary branch at the proximal and middle third of the penis. Therefore, the vascular branches are fan-shaped on the skin and foreskin of the penis. The branch of the superficial dorsal artery and the perforator of the dorsal artery come from a rich vascular network in the penis, distal skin and foreskin (17). The scrotal skin has many hair follicles, and the wear resistance is worse than that of the foreskin. We made the following changes to our surgical procedure. First of all, we sewed between the tunica albuginea and dermis in the 10 and 2 o'clock positions at the penile base, reconstructing the penoscrotal angles and preventing retraction of the corpus penis. Second, to make the reconstructed appearance closer to a normal foreskin, we performed two pedicled skin flaps that were respectively connected with the ventral foreskin and reconstructed with midline symmetric anastomosis on the ventral side of the penis. Finally, we re-covered the corpus penis with a pedicled skin flap, which significantly reduced the tension after the foreskin anastomosis. The foreskin can be maximized to cover the penis that has been completed exposed and this reduces the risk of skin flap ischemia and postoperative scar formation.

Considering the voiding problems, poor hygiene, urinary tract infection, the pessimistic thoughts of patients with the concealed penis, and the parents' anxiety, more researchers suggest that surgery should be completed before puberty (9, 18). We also tend to complete surgery before puberty, but it is not a fatal disease. Therefore, the operation' success depends on the satisfaction of the patients and their parents, and their satisfaction is based on the functional and cosmetic appearance. But there was a significant difference in satisfaction between parents and patients. The overall satisfaction of parents was 97.89%, while it was 75.24% for patients of older children. A possible reason for the lower satisfactory of patients may be the pain and the activity restriction they suffered and their concern for the penile morphology. There also exists a significant difference in their concerns between the penile exposure size and the penile morphology. Parents focus more on the penile exposure size, while patients focus more on the penile morphology. After follow-up, the results showed that the parents were satisfied with the size, morphology, voiding status and hygiene, but the patient's mental health and the damage of surgical treatment have not to be taken seriously. We also found that the patients and their parents not only need a perfected size of the penis, but also a perfected morphology of the penis. The symmetrical pterygoid flap surgery has a significant correction effect for patients with the concealed penis, and the patient's penis is closer to the appearance of the normal foreskin.

There are some disadvantages to our surgical method. First, the surgical method involves a skin transposition flap, and there is a risk of flap infection, flap ischemia and flap necrosis. The complication rate in our case series was 4.42% only; among the cases reported in this group, 7 cases had related complications. Second, this technique is suitable for concealed penis with severe poor exposure; and it has high requirements for surgical techniques. Lastly, the mean follow-up period was months. There is no follow-up regarding sexual intercourse. However, there was no obvious discomfort for postoperative penile erection, and restoration of sexual self-confidence may be useful.

## CONCLUSIONS

The symmetrical pterygoid flap surgery is an effective surgical technique for the management of concealed penis in children producing predictable results and excellent parent and patients' satisfaction. The low rate of complications and good cosmetic outcomes support its use in clinical practice. The postoperative appearance of the penis recovered well. We believe that this surgical method is more effective than the traditional method for selected patients with a concealed penis.

## APPENDIX:

### Supplementary materials Follow-up Table of Concealed Penis.

Pediatric Surgery, Affiliated Hospital of North Sichuan Medical College
